# Age and sex-related comparison of referral-based telemedicine service utilization during the COVID-19 pandemic in Ontario: a retrospective analysis

**DOI:** 10.1186/s12913-023-10373-2

**Published:** 2023-12-07

**Authors:** Hubert Wong, Yasmeen Razvi, Muhammad Akhter Hamid, Niraj Mistry, Guido Filler

**Affiliations:** 1 Department of Pediatrics, Scarborough Health Network, 2867 Ellesmere Rd, Scarborough, Toronto, ON M1E 4B9 Canada; 2 University of Toronto Temerty School of Medicine, Toronto, ON Canada; 3 Hospital for Sick Children, Toronto, ON Canada; 4 Department of Paediatrics, Western University, London, ON Canada

**Keywords:** Telemedicine, COVID-19, Virtual care

## Abstract

**Background:**

The COVID-19 pandemic has led to increased utilization of telemedicine services.

**Methods:**

A retrospective analysis of all referral-based ambulatory telemedicine services in Ontario from November 2019 to June 2021 was collected from the Ontario Health Insurance Plan (OHIP) billing database. Only fee-for-service billings were included in the present analysis. Coincident COVID-19 cases were obtained from Public Health Ontario. Comparisons were made based on age bracket, sex, telemedicine and in-person care.

**Results:**

Billings for telemedicine services in Ontario increased from $1.7 million CAD in November 2019 to $64 million CAD in April 2020 and the proportions reached a mean peak of 72% in April 2020 and declined to 46% in June 2021. A positive correlation was found between the use of telemedicine and COVID-19 cases (p = 0.05). The age group with the highest proportion of telemedicine use was the 10–20-year-olds, followed by the 20–50-year-olds (61 ± 9.0%, 55 ± 7.3%, p = 0.01). Both age groups remained above 50% telemedicine services at the end of the study period. There seemed to be higher utilization by females (females 54.2 ± 8.0%, males 47.9 ± 7.7%, ANCOVA p = 0.05) for all specialties, however, after adjusting for male to female ratio m:f of 0.952:1.0 according to the 2016 census, this was no longer significant.

**Conclusions:**

The use of telemedicine services remained at a high level across groups, particularly the 10–50-year-olds. There were clear age preferences for using telemedicine. Studying these differences may provide insights into how the delivery of non-hospital-based medicine has changed during the COVID-19 pandemic.

## Background

The Coronavirus-19 (COVID-19) pandemic has been a disrupting force to much of society. Healthcare was no exception to this, and substantial changes were made to ensure the delivery of services while enabling physical distancing to minimize the risk of viral spread [1].

Telemedicine and new technologies have gained substantial importance in the management of chronic disease [2–4]. Telemedicine has been key to enabling the continuity of ambulatory medical services even at the height of the pandemic, as its use rose dramatically [5, 6]. In the province of Ontario, virtual visits increased from 1.6% in the summer of 2019 to 70.6% in the summer of 2020, with a similar increase in the number of physicians offering multiple telemedicine visits per year [5]. The use of telemedicine appears to be widespread across multiple services, and regions, with favourable reception among families [5–10].

Descriptions, regarding the adoption of telemedicine by age and sex, has been more limited [5, 11, 12]. Most reports describe local, institutional, or specialty-based experiences. Some reports have discussed age-specific pediatric experiences across a variety of subspecialties ranging from emergency medicine to pediatric nephrology and pain management [13–15]. Bhatia et al. describe a cross-sectional study of telemedicine use in the pre-pandemic period until just after the onset of the pandemic [5]. They noted age and sex variations in the proportion of the population with at least one virtual visit during this period [5].

We hypothesized that there were age and sex-related differences in the adoption of telemedicine services during the pandemic with the youngest age groups likely showing the highest proportion of telemedicine services and with services likely showing a dramatic adoption in the earliest stages of the pandemic with a gradual decline in adoption over the course of the study itself. We aim to describe these factors, with the goal of helping us identify barriers to telemedicine services that will help guide future policy and resource considerations in trying to reduce inequities within the healthcare system.

## Methods

### Data collection

This study was a retrospective analysis of the delivery of referral-based ambulatory telemedicine services within the Canadian province of Ontario. This area represents a total population of 13.5 million and a population of 3.02 million children 19 years and younger [16]. Provincially funded public healthcare is widely available to all Canadian citizens and services are claimed by physicians through a single insurance provider, the Ontario Health Insurance Plan (OHIP). Temporary, telemedicine claims were introduced by OHIP on March 14, 2020, and these same service claims remained unchanged during the study period [17].

**Fig. 1:**
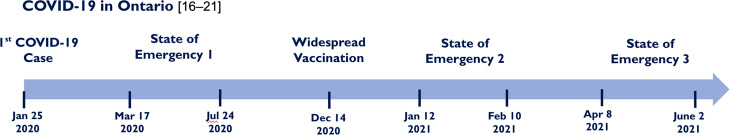
Timeline of public health announcements and vaccine response to covid-19 pandemic in Ontario

COVID-19 case data was obtained from Public Health Ontario’s publicly available records from January 15, 2020, until June 30, 2021 [18]. The province of Ontario reported the first case of COVID-19 on January 25th, 2020, and it experienced varying degrees of restrictions and lockdowns throughout the pandemic, which were applied inconsistently. There were three states of province-wide emergencies. These were from March 17 to July 24, 2020; January 12 to February 10, 2021, and April 8 to June 2, 2021 [19–24]. Widespread vaccination began on December 14, 2020 (Fig. 1). Ontario is the most populous province in Canada, representing 34% of the total population of Canada. Each province has their own health insurance plans, which means coverage varies from place to place. All citizens and permanent residents can receive medically necessary physician and hospital services for free, but coverage of other services will depend on where residency is located. For the case of telemedicine, all provinces adopted that. [25] The data may not be generalizable for the country.

Detailed search criteria were extracted from the OHIP claims database by Ontario Medical Association (OMA) Economics, Research & Analytics between February 9, 2022, and March 29, 2022, regarding healthcare services from the period November 2019 until June 2021. Claims were primarily physician services submitted to OHIP and represent the most accurate information available on Ontario services. The start date included a baseline period from November 2019 until February 2020 prior to the pandemic for comparison with data from the post pandemic period. The end date of the study period was chosen based on when complete information was still available for analysis. Data represents all fee-for-service physician billings in the Province of Ontario for this period.

Specific data included monthly billing patterns among all referral-based ambulatory services, including cardiology, community medicine, clinical immunology, critical care, dermatology, endocrinology, gastroenterology, genetics, geriatrics, haematology, infectious disease, internal and occupational medicine, nephrology, medical oncology, neurology, paediatrics, physical medicine, psychiatry, radiation oncology, respirology, and rheumatology. Our focus was to understand the changes in the delivery of outpatient medical services during the pandemic. Hospital-based care was not included in the scope of services we were focused on analyzing.

Only professional fee for service billings were included. We excluded other alternative payment models such as shadow billings, technical billings, alternative funding plans or salaries. Telephone and virtual video visits were not distinguished or analyzed separately. All surgical, radiology, obstetrics, anaesthesia, emergency, acute care, and family medicine services were excluded. Surgical, radiology, anaesthesia and obstetrics were excluded because their services are less compatible with referral-based ambulatory services. Family practice and general practice medicine were also excluded to prevent comparisons of telemedicine usage in medical specialties that are largely referral based to family and general practice that are typically self-referred.

Monthly service claims were then categorized to two groups: (1) Telemedicine Professional Services and Non-Telemedicine Professional Services and (2) the proportion of Telemedicine Professional Services to All Professional Services were also reported. Comparisons were then made based on age bracket and sex. Ten age brackets were used based on each decade of life. These age brackets were 0–10 years old, 10–20 years old, 20–30 years old, 30–40 years old, 40–50 years old, 50–60 years old, 60–70 years old, 70–80 years old, 80–90 years old and 90 + years of age. The age brackets with the highest population were the 20–30-year-old followed by the 30–40-year-old and 50–60-year-old age brackets. Each of these age brackets had a population of 2 million or more in 2021 [13]. To calculate the mean adoption of telemedicine users per age bracket, for each age bracket, total telemedicine services over the 15-month period measured during the COVID-19 pandemic were divided by total health care services over that same period. The result is reported as the mean adoption of telemedicine services over the 15-month period from April 2020 until June 2021.

### Statistical analysis

Wherever possible, we used simple descriptive statistics after the determination of normal distribution with the Shapiro-Wilk test for continuous data. Parametric data were expressed as mean ± one standard deviation, and non-parametric data were expressed as median and range. Means for healthcare services were calculated based on the period after the start of the pandemic from April 2020 until June 2021. Comparisons using two sets of parametric data were assessed using the two-tailed t-test or Pearson correlation. Comparison of non-parametric data was accomplished with the Mann-Whitney U Test or Spearman correlation. Regression analysis for the proportion of virtual care for each age bracket over the study period was transformed to linear datasets for comparison of means using Analysis of covariance. In all circumstances, a p-value less than 0.05 was considered statistically significant.

## Results

Data reflecting total healthcare services in referral-based ambulatory services after the start of the pandemic were normally distributed. Non-telemedicine professional services were also normally distributed but skewed in a way that did not reach statistical significance. Monthly COVID-19 case numbers were not normally distributed.

### Telemedicine trends for referral-based ambulatory telemedicine services

**Fig. 2:**
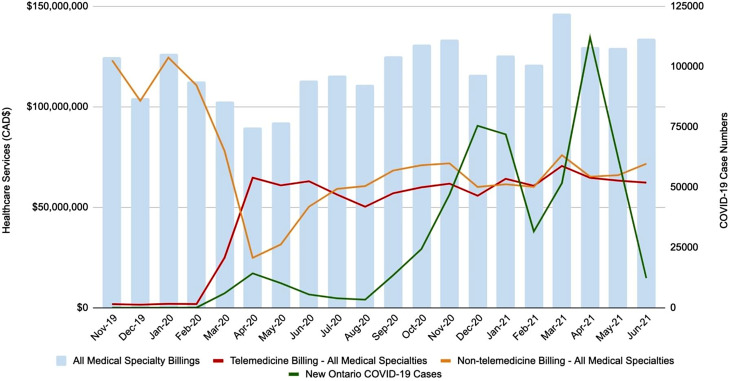
Distribution of non-hospital based healthcare services in comparison with covid-19 case numbers from November 2019 until June 2021. All medical specialty billing, telemedicine billings- all medical specialties, non telemedicine billings- all medical specialties, new Ontario COVID-19 cases

Data for telemedicine and non-telemedicine services for all referral-based ambulatory services, along with monthly Ontario COVID-19 cases, are summarized in Table 1; Fig. 2. There was a rise in telemedicine services for all medical professionals from approximately $1.8 million CAD in November 2019 to $64.7 million CAD in April 2020. During the same period, monthly non-telemedicine services dropped from $123 million CAD at baseline to $24 million CAD in April 2020. Monthly non-telemedicine billings have increased since April 2020, while monthly telemedicine billings remained consistent with average monthly telemedicine services of $61 ± 4.8 million CAD. The overall proportion of telemedicine billings in comparison declined from 72% of total services in April 2020 to 46% in June 2021. Healthcare services have since surpassed pre-pandemic levels, going from $125 million CAD in November 2019 to $134 million CAD in June 2021 (Table 1; Fig. 2). There was a positive correlation between the use of virtual care and COVID-19 case numbers both before and after the first COVID-19 pandemic wave (Spearman p = 0.05). There is a negative correlation between non-telemedicine services and COVID-19 case numbers from before the pandemic, but this relationship is not significant after the first wave (Spearman p = 0.05).

**Table 1:** Comparison of telemedicine and non-telemedicine health services with COVID-19 case numbers in the Province of Ontario from November 2019 until June 2021. Mean and SD are based on the period from April 2020 until June 2021

Month	Telemedicine Billing - All Referral based Medical Specialties	Non-telemedicine Billing - All Referral Based Medical Specialties	All Referral Based Medical Specialty Billings	Proportion of Billings as Telemedicine (%)	New Ontario COVID-19 Cases
Nov-2019	$1,753,641	$123,062,345	$124,815,986	1.40%	0
Dec-2019	$1,476,017	$102,910,701	$104,386,718	1.41%	0
Jan-2020	$1,937,928	$124,453,391	$126,391,319	1.53%	9
Feb-2020	$1,810,633	$110,673,452	$112,484,085	1.61%	49
Mar-2020	$24,866,303	$77,928,857	$102,795,160	24.19%	5970
Apr-2020	$64,681,956	$24,808,350	$89,490,306	72.28%	14,234
May-2020	$60,871,300	$31,475,116	$92,346,416	65.92%	10,149
Jun-2020	$62,899,066	$50,270,489	$113,169,555	55.58%	5479
Jul-2020	$56,298,432	$59,095,723	$115,394,155	48.79%	3877
Aug-2020	$50,233,344	$60,497,329	$110,730,673	45.37%	3343
Sep-2020	$56,958,867	$68,248,860	$125,207,727	45.49%	13,504
Oct-2020	$59,906,384	$70,884,774	$130,791,158	45.80%	24,339
Nov-2020	$61,705,546	$71,826,162	$133,531,708	46.21%	46,942
Dec-2020	$55,696,528	$60,082,522	$115,779,050	48.11%	75,430
Jan-2021	$64,145,018	$61,406,159	$125,551,177	51.09%	71,841
Feb-2021	$60,626,757	$60,131,057	$120,757,814	50.21%	31,578
Mar-2021	$70,523,391	$75,879,558	$146,402,949	48.17%	51,646
Apr-2021	$64,606,158	$65,220,706	$129,826,864	49.76%	112,059
May-2021	$63,208,660	$65,921,725	$129,130,385	48.95%	62,087
Jun-2021	$62,225,610	$71,612,765	$133,838,375	46.49%	12,262
Mean	$60,972,468	$59,824,086	$120,796,554	51.21%	
SD	$4,791,775.17	$14,454,623.91	$15,316,341.17	7.82%	

### Age analysis

**Fig. 3:**
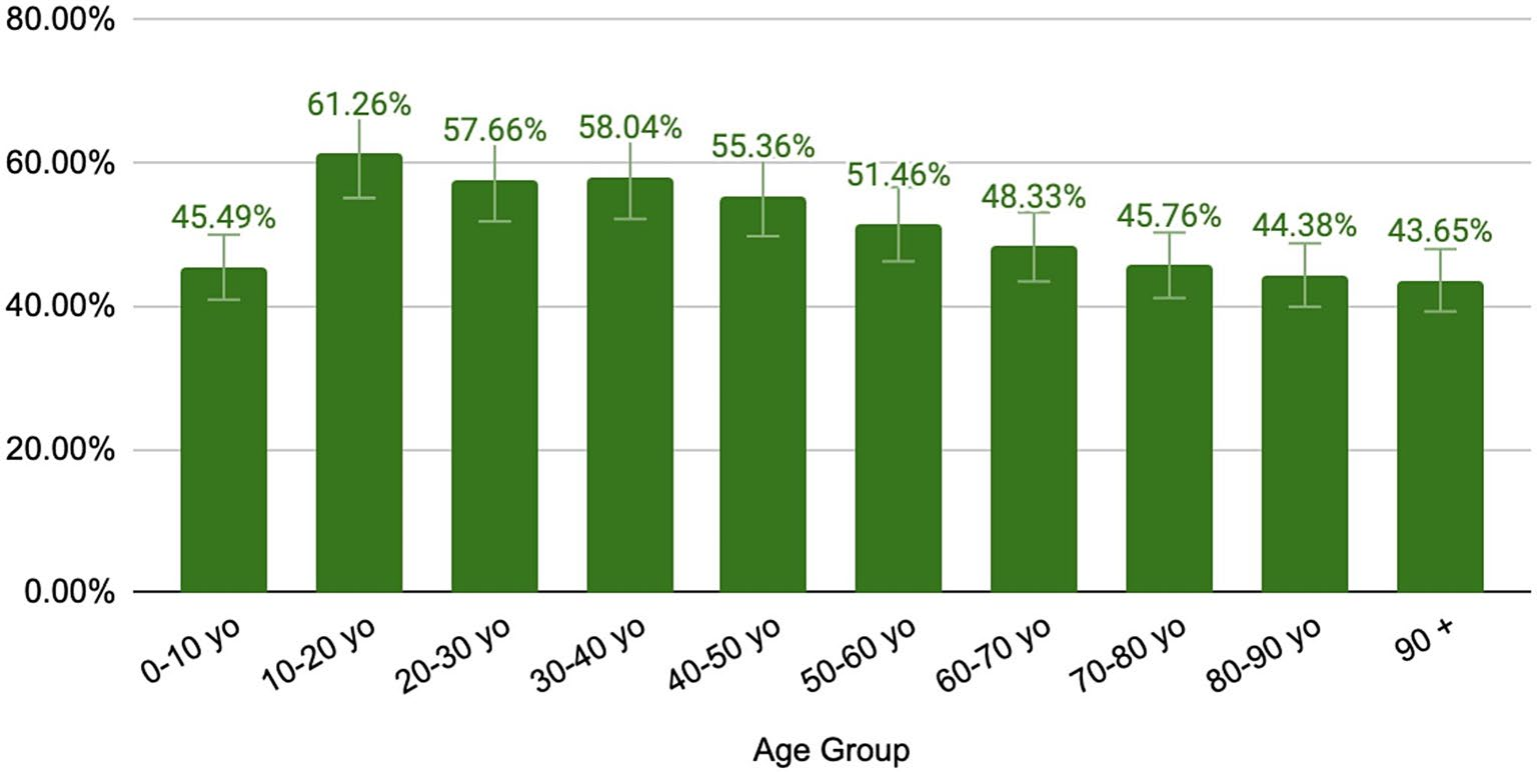
Mean adoption of telemedicine services according to age group

The age bracket with the highest rate of use of telemedicine services is the 10-20-year-olds, which peaked at 83.2% in April 2020 (just after the start of the pandemic), before reaching a mean of 61.26% (± 8.92%) in the final 15 months of the study. This is followed by a slightly lower level of use for each successive age bracket (Table 2; Fig. 3). There is a more precipitous decline in the use of telemedicine following the 60-70-year-old age bracket. The youngest age bracket (0-10-year-old) had a similar rate of use as the 70 to 80-year-old bracket (45.5 ± 7.0%, 45.76 ± 8.67%). The age brackets with the highest use of telemedicine in CAD are the 50 to 60 and 60-70-year-old brackets (9.6 ± 0.7, 9.8 ± 0.8 in millions CAD). This is consistent with data both prior to and during the onset of the pandemic that showed the 60–70-year-old age group had the highest average monthly health care services utilized (19.0 ± 1.9 and 20.7 ± 2.8 in millions CAD respectively).

**Table 2:** Comparison of age bracket with health care services and COVID-19 case numbers from November 2019 until June 2021. The age bracket 10–20-year-old have the highest mean rate of telemedicine service at 61.3 ± 8.9%. Each subsequent age bracket has a declining rate of telemedicine use although the overall proportion of telemedicine services remains high (51.1 ± 9.7%)

Month	Proportion of Billings as Telemedicine 0–10(%)	Proportion of Billings as Telemedicine 10–20(%)	Proportion of Billings as Telemedicine 20–30(%)	Proportion of Billings as Telemedicine 30–40(%)	Proportion of Billings as Telemedicine 40–50(%)	Proportion of Billings as Telemedicine 50–60(%)	Proportion of Billings as Telemedicine 60–70(%)	Proportion of Billings as Telemedicine 70–80(%)	Proportion of Billings as Telemedicine 80–90(%)	Proportion of Billings as Telemedicine 90+(%)	New Ontario COVID-19 Cases
Nov-2019	0.48%	1.08%	2.63%	2.77%	2.15%	1.59%	1.19%	0.83%	0.59%	0.38%	0
Dec-2019	0.50%	1.12%	2.69%	2.94%	2.24%	1.51%	1.16%	0.80%	0.57%	0.50%	0
Jan-2020	0.58%	0.91%	2.97%	3.09%	2.48%	1.69%	1.27%	0.85%	0.65%	0.63%	9
Feb-2020	0.70%	0.90%	3.03%	3.28%	2.48%	1.76%	1.27%	0.92%	0.65%	0.74%	49
Mar-2020	22.85%	28.59%	27.14%	28.20%	26.80%	24.14%	22.43%	21.31%	19.96%	17.54%	5970
Apr-2020	64.45%	83.21%	72.93%	73.72%	75.63%	72.96%	71.28%	70.08%	68.73%	65.12%	14,234
May-2020	58.29%	78.97%	69.54%	70.09%	69.96%	67.21%	63.94%	61.14%	59.04%	56.89%	10,149
Jun-2020	49.56%	68.11%	61.61%	62.13%	59.64%	56.28%	52.83%	49.40%	47.93%	49.91%	5479
Jul-2020	43.32%	59.89%	55.75%	56.35%	53.52%	48.97%	45.91%	42.26%	41.26%	41.46%	3877
Aug-2020	40.09%	54.93%	52.61%	53.54%	50.44%	45.64%	42.10%	39.24%	37.77%	36.95%	3343
Sep-2020	41.45%	54.70%	53.83%	53.57%	50.50%	45.79%	42.03%	39.20%	37.60%	38.16%	13,504
Oct-2020	40.03%	53.99%	53.09%	53.57%	50.70%	46.04%	42.81%	40.21%	39.32%	39.08%	24,339
Nov-2020	39.84%	54.31%	53.51%	54.14%	50.70%	46.77%	43.04%	40.58%	39.73%	39.81%	46,942
Dec-2020	42.99%	57.19%	53.87%	55.57%	51.85%	48.19%	44.93%	42.57%	41.79%	40.71%	75,430
Jan-2021	46.16%	62.91%	57.42%	57.32%	54.55%	50.51%	47.81%	46.01%	44.74%	45.44%	71,841
Feb-2021	45.38%	60.33%	57.16%	56.81%	53.36%	49.81%	47.05%	44.90%	43.88%	44.33%	31,578
Mar-2021	42.43%	57.09%	56.13%	55.66%	52.06%	47.97%	45.07%	42.75%	41.15%	40.34%	51,646
Apr-2021	44.46%	59.63%	57.10%	56.79%	53.72%	49.47%	46.88%	44.08%	43.02%	41.20%	112,059
May-2021	43.67%	58.59%	56.02%	56.39%	52.89%	49.11%	45.85%	43.31%	41.34%	39.37%	62,087
Jun-2021	40.30%	55.04%	54.36%	54.89%	50.88%	47.14%	43.37%	40.69%	38.46%	36.04%	12,262
mean	45.49%	61.26%	57.66%	58.04%	55.36%	51.46%	48.33%	45.76%	44.38%	43.65%	
SD	7.07%	8.92%	5.99%	6.06%	7.53%	8.07%	8.41%	8.67%	8.58%	8.00%	

### Analysis of sex-based differences

**Fig. 4:**
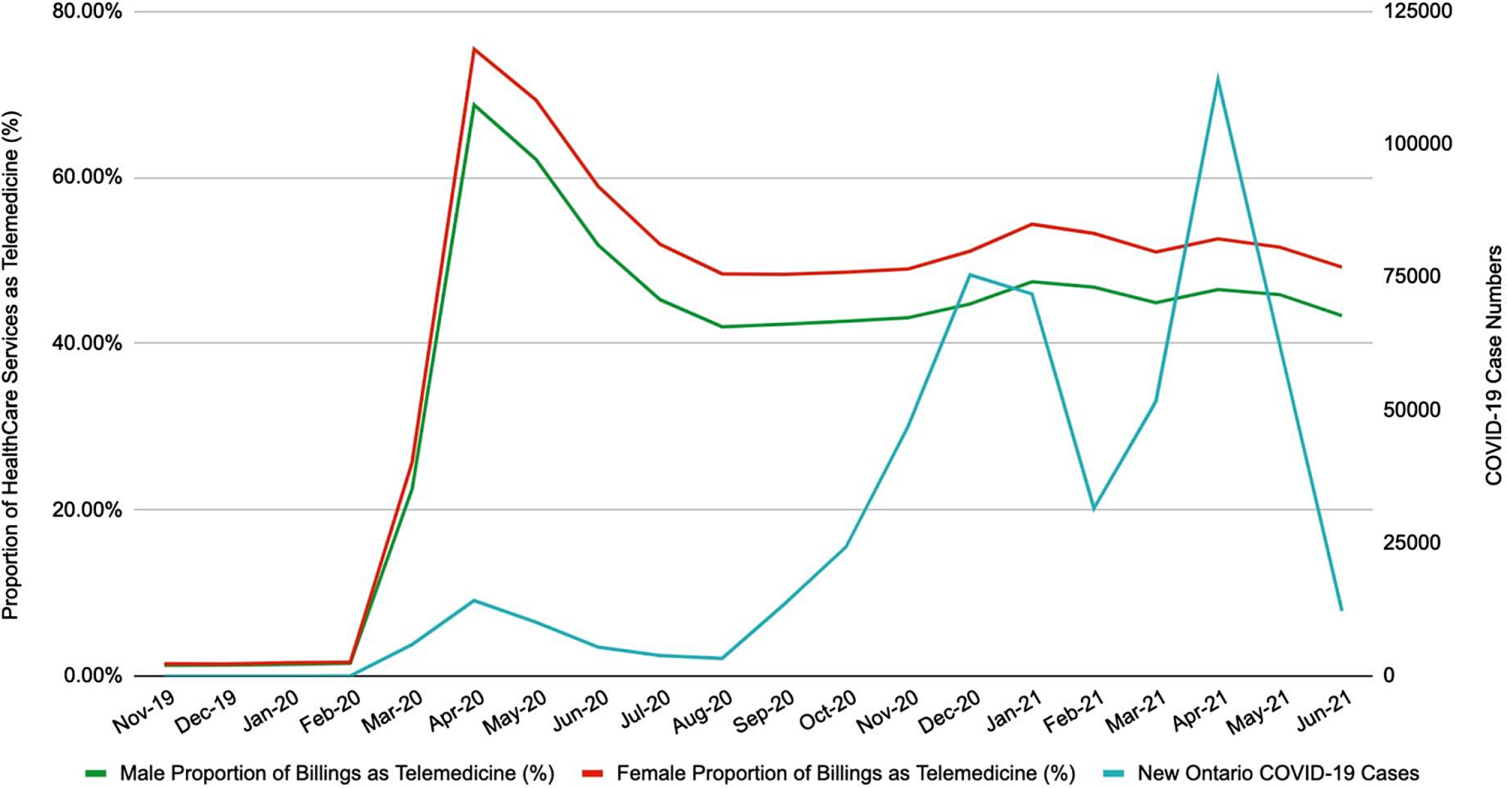
Comparison of gender with telemedicine use and COVID-19 case numbers from November 2019 until June 2021. Male proportion of billings as telemedicine (%), Female proportion of billings as telemedicine (%), new Ontario COVID-19 cases

Females have higher use of healthcare services overall compared to males (female 64.3 ± 8.4, males 56.4 ± 6.9 in millions CAD, p = 0.01). They also have a higher rate of use of telemedicine when adjusted for total healthcare services (54.2 ± 8.0%, 47.9 ± 7.7%, ANCOVA p = 0.05). In April 2020, the use of telemedicine services peaked among females at 75.43% (Table 3; Fig. 4).

**Table 3:** Comparison of sex with healthcare services and COVID-19 case numbers from November 2019 until June 2021. Females have a higher proportion of telemedicine services compared to males (females 54.2 ± 8.0%, males 47.9 ± 7.7%, ANCOVA p = 0.05)

Month	Male Telemedicine Billing - All Referral Based Medical Specialties	Male All Referral Based Medical Specialty Billings	Female Telemedicine Billing - All Referral Based Medical Specialties	Female All Referral Based Medical Specialty Billings	Male Proportion of Billings as Telemedicine (%)	Female Proportion of Billings as Telemedicine (%)	New Ontario COVID-19 Cases
Nov-2019	$786,576	$60,042,574	$967,065	$64,773,412	1.31%	1.49%	0
Dec-2019	$691,750	$51,132,548	$784,267	$53,254,170	1.35%	1.47%	0
Jan-2020	$874,151	$61,195,873	$1,063,777	$65,195,446	1.43%	1.63%	9
Feb-2020	$842,667	$54,543,759	$967,966	$57,940,326	1.54%	1.67%	49
Mar-2020	$11,204,149	$49,671,038	$13,662,154	$53,120,644	22.56%	25.72%	5970
Apr-2020	$28,810,518	$41,918,014	$35,871,438	$47,554,344	68.73%	75.43%	14,234
May-2020	$26,968,462	$43,393,522	$33,902,838	$48,927,842	62.15%	69.29%	10,149
Jun-2020	$27,653,052	$53,314,839	$35,246,014	$59,822,665	51.87%	58.92%	5479
Jul-2020	$24,628,792	$54,391,345	$31,669,640	$60,971,016	45.28%	51.94%	3877
Aug-2020	$21,978,886	$52,307,455	$28,254,458	$58,387,174	42.02%	48.39%	3343
Sep-2020	$24,957,916	$58,952,517	$32,000,951	$66,211,733	42.34%	48.33%	13,504
Oct-2020	$26,184,635	$61,341,197	$33,721,749	$69,411,709	42.69%	48.58%	24,339
Nov-2020	$26,928,036	$62,476,843	$34,777,510	$71,013,999	43.10%	48.97%	46,942
Dec-2020	$24,412,580	$54,538,275	$31,283,947	$61,200,720	44.76%	51.12%	75,430
Jan-2021	$27,934,682	$58,894,701	$36,210,336	$66,610,241	47.43%	54.36%	71,841
Feb-2021	$26,456,537	$56,552,921	$34,170,219	$64,167,016	46.78%	53.25%	31,578
Mar-2021	$30,457,052	$67,809,663	$40,066,339	$78,535,870	44.92%	51.02%	51,646
Apr-2021	$27,899,597	$59,988,458	$36,706,561	$69,789,014	46.51%	52.60%	112,059
May-2021	$27,321,357	$59,535,700	$35,887,303	$69,543,423	45.89%	51.60%	62,087
Jun-2021	$26,763,160	$61,715,702	$35,462,450	$72,083,375	43.37%	49.20%	12,262

However, the gender ratio of males to females was 95.2 males to 100 females in the 2016 census, not significantly different from the previous census (95.1). We analyzed the healthcare utilization before the pandemic based on the time frame from November 2019 to February 2020, when only very few COVID-19 cases occurred. These billings for females were significantly higher per month, both for all medical specialty billings (p = 0.0076, paired t-test) and telemedicine billings (p = 0.0078, paired t-test). When adjusting these ratios by the gender ratio, there was no gender difference in all medical specialty billings (p = 0.2141, paired t-test) whereas it remained significant for telemedicine specialty billings (p = 0.0041, paired t-test). As such, the gender differences by sex preceded the pandemic.

## Discussion

The Coronavirus disease 2019 (COVID-19) pandemic resulted in a significant transformation in the delivery of medical services. Immediately after the onset of the pandemic, telemedicine services were observed to have significantly increased in response to COVID-19 cases. The findings in our paper are consistent with literature published from other Canadian provinces, the United States of America (USA), and Europe on the early use of telemedicine after the start of the pandemic [5–7, 26–28]. To the best of our knowledge, this study represents the longest period following the start of the pandemic that monitored referral-based ambulatory telemedicine services and their continuity of service delivery over that time. We have also further explored and demonstrated a persistent age and sex preference for adopting telehealth services that continued through the initial three waves of the pandemic over a course of fifteen months. We found no sex differences in health care billing per month for all medical specialties pre-pandemic whereas there was a significant sex difference for specialty telemedicine with higher utilization among females already pre-pandemic.

Our study specifically identified the impact of telemedicine on referral-based ambulatory services where the impact of telemedicine was likely to be the greatest for both the clinicians and the families. Our analysis excluded general practice and family-based medicine mainly due to the difficulty in comparing medical specialties which are typically referral based with primary care practice. In addition, the effect of the pandemic and the use of telemedicine on primary care practice has already been well-reported elsewhere [29, 30].

A similar study conducted by Bhatia et al., which represents the closest contextual analysis of telemedicine in the province of Ontario, demonstrated age and sex-related differences in telemedicine use primarily in the pre-pandemic period [5] and compared to our study, they included all medical services in their assessment of the use of telemedicine such as emergency medicine, obstetrics, surgery, intensive care, hospital-based medicine, anaesthesia, and radiology; and not all these services may be suitable to a telemedicine platform [5]. Furthermore, Bhatia et al. used at least one virtual visit as their primary outcome measure compared to our study which measured total healthcare services [5]. Using the billing as a surrogate of the financial impact for healthcare services may under-report the proportion of telemedicine services being conducted in non-hospital-based clinics in older age brackets. This may occur because of the routine use of non-hospital-based medical procedures that are common surveillance tools among older age patients compared to younger age ones. Examples of these procedures include colonoscopies, pulmonary function test, polysomnography, and a variety of cardiac diagnostic testing [31, 32].

There are important similarities and differences that can be noted between the two studies. For example, Bhatia et al. noted that the highest overall use of telemedicine occurred within 18-49-year-old patients [5]. Conversely, our study noted that this occurred in the 50-70-year-old group, which is consistent with their higher overall use of healthcare in general [33]. For Bhatia et al., the age group with the highest proportion of telemedicine services was reported as the 65–79-year age group (72%) with under 18 years having the lowest proportion of telemedicine services (40%) [5]. In contrast, we noted that the age bracket with the highest proportion of telemedicine services was in fact in the 10–20-year group, with each subsequent age bracket declining in the proportion of telemedicine services. Our findings are consistent with general expectations as there is substantial evidence that shows higher use of electronic mobile devices, social media, and online shopping in younger age groups [34–36]. We also note that in most circumstances, it is not the child that determines their own healthcare services. These decisions are often made by their family and do not always reflect the age of the patient, although the age of the patient may still reflect on the suitability for telemedicine services. In a qualitative study from the United States of America, caregivers of children welcomed the use of telemedicine because of prevention of exposure to COVID-19, but found lack of in-person interactions, fear of compromised confidentiality, and the potential for misdiagnosis to be a barrier. [37] Furthermore, an important distinction between our study and Bhatia et al. is the exclusion of primary care from the current analysis, whereas Bhatia et al. included all services.

This study shows a steep decline in the proportion of telemedicine services being delivered after the first wave of the pandemic, but this proportion remains relatively stable during the second and third waves and persists during periods when the state of emergency is lifted. In fact, we note that the average monthly use of telemedicine remains very stable from April 2020 until June 2021; the main determining factor that affects the proportion of telemedicine use is the resumption of non-telemedicine services that recovered following the first wave. The Mayo Clinic reported a different experience in pediatric telemedicine showing a decline in telemedicine care of 66% following the lifting of stay-at-home orders [28].

Bhatia et al. reported that females had higher overall use of telemedicine (56.6% v. 49.4% p < 0.001), like our results. This difference persists throughout the fifteen months following the start of the pandemic, although it is unclear why this finding exists. Females have a higher use of healthcare services overall, but even when accounting for this higher utilization, the proportion of telemedicine services used by females exceeds that used by males. This difference may occur because of greater barriers for females to access in-person healthcare. These barriers may include a higher female unemployment rate and a higher involvement in childcare and online schooling during the pandemic [38, 39]. Several authors reported variations in healthcare use by sex, with some studies reporting a higher utilization of healthcare resources by females [11, 31, 40, 41]. However, after adjusting for the 2016 male/female ratio, in our study the higher utilization of telemedicine was only seen for specialty telemedicine and predated the pandemic. Similar observations were made by Javier-DesLoges [42] in California, USA. The authors argued that these differences may compound existing disparities in care and that they were not associated with provider attributes. Multivariable logistic regression analysis revealed that individuals with Hispanic race, older age or with Medicaid-only insurance were significantly less likely to access telemedicine. The reasons are not fully understood, but access to technology and computer literacy may play a role. These findings may also vary by region and culture. In a retrospective study in Bangladesh, male patients had a higher dependency on telemedicine than females [43].

### Knowledge gaps and future directions

The results of the present study indicate a sex and age preference for adopting telemedicine, both pre and during the pandemic. While this may indicate preferences in care, it may also suggest potential barriers for groups who accessed telemedicine to a lesser degree [42, 44]. These may include inequities in the availability of providers and technology, as well as variations in health and technological literacy. To better appreciate barriers to accessing telemedicine, future work should qualitatively explore these experiences [45]. Interestingly, a recent study found that adolescents disliked telemedicine more during the pandemic than pre-pandemic and wanted in-person visits more often [46]. Furthermore, research should continue to assess patient and provider satisfaction with telemedicine and correlate this with long-term outcomes [15]. Obstacles associated with telemedicine consultations such as limited physical examinations and their effects on diagnostic determination should be reviewed moving forward [42]. Finally, the application of telemedicine to hospital-based care and opportunities for its use should be explored.

The widespread use of telemedicine services has considerable potential, with broad social, economic, and environmental implications. Telemedicine has proved adept at allowing the healthcare system to circumvent large, societal disruptions. Notwithstanding, there is a need to evaluate the efficacy, as some growing literature suggests that the quality of care is not equivalent [47, 48]. It is also important to evaluate the differing views of caregivers and minors, which have been shown to be quite different in caregivers and adolescents [9].

### Limitations

Limitations of the present study include the restriction of data to the province of Ontario, which serves a large and heterogeneous population. As such, the experience in Ontario may not be generalizable to other geographic areas. Furthermore, a limitation is our reliance on OHIP data as the sole measure of healthcare usage, without corroboration from other data sources. We also note our exclusion of alternative fee structures from our analysis apart from the fee-for-service model that was included. There are two issues that complicate the inclusion of these alternate fee structures within our analysis. First, most of the alternate fee structures are based on block payments for either large groups of services or salaries and therefore cannot be analysed separately for telemedicine and non-telemedicine services. Second, the basic assumptions used to analyse non-hospital-based medical services may not be applicable to these models of care. Excluding these other forms of payments for healthcare services does however limit the generalizability of our present study as traditional fee-for-service accounts for just more than 55% of total healthcare services in the Province of Ontario [49].

## Conclusions

Overall, the use of telemedicine services significantly increased during the pandemic for all referral-based ambulatory services. This rise in telemedicine was positively associated with monthly COVID-19 case counts, indicating that telemedicine served to enable continued ambulatory care while promoting physical distancing. The use of telemedicine remained consistently elevated 15 months into the pandemic. There was an age difference associated with telemedicine, as individuals aged 10–20 had the highest proportion of telemedicine use. Future areas of work should seek to understand potential barriers in access to telemedicine services for subpopulations with a lower uptake, as well as long-term outcomes associated with telemedicine. Furthermore, the potential for telemedicine to allow for healthcare system adaptation to future societal stressors such as climate change and the economy should be further explored.

## Data Availability

The datasets used and/or analysed during the current study are available from the corresponding author on reasonable request.
